# Optimal stress and deformation partition in gradient materials for better strength and tensile ductility: A numerical investigation

**DOI:** 10.1038/s41598-017-10941-7

**Published:** 2017-09-08

**Authors:** Yao Wang, Guangxue Yang, Wenjing Wang, Xi Wang, Qiang Li, Yujie Wei

**Affiliations:** 1 Key Laboratory of Vehicle Advanced Manufacturing, Measuring and Control Technology (Beijing Jiaotong University), Ministry of Education, Beijing Jiaotong University, Beijing, 100044 China; 2 State Key Laboratory of Nonlinear Mechanics (LNM), Institute of Mechanics, Chinese Academy of Sciences, Beijing, 100190 China

## Abstract

Inspired by recent progress in developing gradient materials with excellent performances, here we report a systematic finite-element based investigation to show how the strength and tensile ductility of gradient crystalline metals depend on their microstructure characteristics. We reveal that the yielding strength of polycrystalline metals with gradient grain size can be significantly enhanced at no reduction in ductility. By employing a representative 3D voronoi gradient sample, we demonstrate that the redistribution of stress and deformation in the gradient structure - stronger grains carry more load and ductile ones share more deformation - accounts for the realized optimal property in strength and ductility. In addition, the hardenability of the ductile domain is beneficial to circumvent pre-mature plastic instability in gradient samples.

## Introduction

Gradient materials that are inspired from the intelligence of nature^1–3^, with microstructures varying from the surface to the core of a sample, have received increasing attention due to their excellent mechanical performances. In contrast to their homogeneous counterparts, gradient structures owe enhanced mechanical properties, including better wear resistance, low friction, high strength and ductility^4–10^. So far, a vast number of experimental and theoretical researches have been dedicated to reveal how a gradient microstructure is prepared and how the unique gradient microstructures lead to the extraordinary mechanical properties. Lu^4^ and Fang *et al*.^5, 6^ used surface mechanical grinding treatment (SMGT) method to prepare a nano-grained (NG) Cu film with a spatial gradient in grain size on a core coarse-grained (CG) Cu substrate. Such samples have a tensile ductility comparable to that of the CG counterpart but much higher yielding strength. In gradient samples prepared by surface mechanical attrition treatment (SMAT), Wu *et al*.^8^ observed the same type of strengthening without apparent reduction in ductility. Wei *et al*. employed simple pre-torsion to both twin induced plasticity steel^9^ and the most broadly used 304 austenitic steel^10^. It was revealed that the gradient deformation imposed by pre-torsion leads to gradient twin densities in those materials which are easy to be twinned at room temperature. Such pre-existing twin density and twin size evade the strength-ductility trade-off dilemma commonly seen in steels, and also enhance the fatigue resistance of 304 austenitic steel^10^. When employing the same strategy to AZ31B, a magnesium alloy which dominantly hexagonal centred cubic structure, Liu and Wei^11^ realized gradient twin spacing. Such twin gradient reduces the yield-asymmetry in as-received AZ31B, much higher compressive yielding strength than that of tension, and gave rise to reversible torsional plasticity driven by the gradient twin structure.

Theoretically, Lee *et al*.^12^ analyzed the generalized stress and strain relationship in samples with grain size gradient. Using finite element method, Li and Soh^13, 14^ modeled the strengthening effects in samples containing grains ranging from tens of nanometers to tens of micrometers. More recently, Zeng *et al*.^15^ used finite element based crystal plasticity constitutive model to understand the plastic deformation partition in a two-dimensional (2D) gradient sample. Their simulations elegantly show that there exist a strain gradient in those gradient samples, in consistent with experimental observations by Wu *et al*.^8^.

It is worth noting that previous work is associated with small deformation in those samples which could actually deform largely. It would be of interest to examine the gradient effect at large deformation. In addition, while aforementioned work demonstrates clearly the strengthening effect due to the presence of gradient structures, it remains unclear how those gradient would impact the tensile ductility of the materials, as the latter is equally important, if not more, as the strength of materials. As we are examining the geometrical effects of grains and real structures are three-dimensional, we wonder whether 3D simulations would add more information to existing 2D analysis. Motivated by those factors, we report a finite-element based theoretical analysis to show how the yielding strength of the gradient structure can be significantly enhanced at no reduction in ductility in representative 3D voronoi gradient microstructure.

## Structure and material preparation

**Figure 1:**
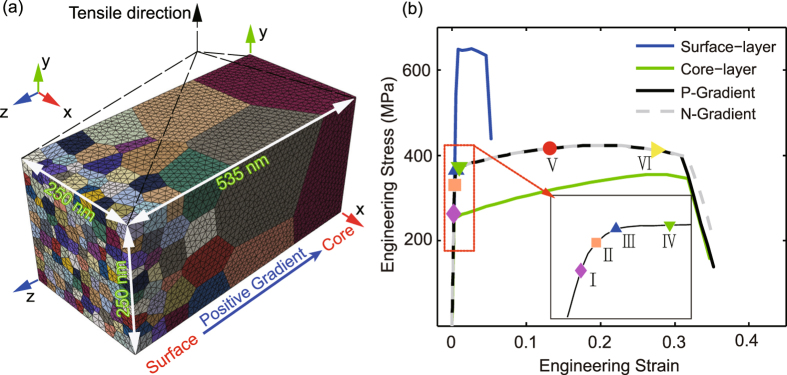
Three-dimensional voronoi grains with gradient in grain size. (**a**) The dimensions and boundary conditions applied to the three-dimensional gradient element model with gradient polycrystalline structure. It consists 221 grains and has grain size from 25 *nm* to about 250 *nm* along the z-axis. (**b**) Quasi-static axial tensile engineering stress-strain curves for the three-dimensional gradient grain size copper. Positive gradient (P-Gradient for short) is a gradient structure with the surface of grain size 25 *nm* and the core of grain size 250 *nm*. Negative gradient (N-Gradient for short) is a gradient structure with the surface of grain size 250 *nm* and the core of grain size 25 *nm*, the enlarged image for certain positions is shown in the figure. Six representative applied strains are marked in this figure, point I to VI are the applied true strain 0.25%, 0.38%, 0.54%, 0.98%, 12.36% and 24.31%, respectively.

In order to capture the stress-strain response from the simulation accurately, we conduct a series of FEM simulations with grain-level topological information from real microstructure. All of the polycrystalline information is determined by the voronoi tessellation modules integrated in the Python programming language. A typical meshed 3D sample is presented in Fig. 1a.

For the purpose of obtaining both the strength and the tensile ductility of gradient polycrystalline Cu under uniaxial tension, we choose a constitutive model which considers the failure of material for the 3D model. There exist many constitutive models which are capable of capturing the whole deformation process involving plasticity, void nucleation, void growth, coalescence, and macroscopic fracture till material failure^16–23^. For convenience, we adopt the GTN model (Gurson-Tvergaard-Needleman)^16–20^ embedded in ABAQUS^24^ to capture both the hardening progress and the softening progress of the material. The plastic potential in the GTN model^24^ is given as

1 $$\phi ={(\frac{{\sigma }_{e}}{{\sigma }_{0}})}^{2}+2{q}_{1}f\cdot ch(\frac{3{q}_{2}{\sigma }_{m}}{2{\sigma }_{0}})-1-{q}_{3}{f}^{2}$$

where ${\sigma }_{e}$ and ${\sigma }_{m}$ are the effective (von Mises) stress and the hydrostatic tension of a representative volume element, ${\sigma }_{0}$ is the initial yielding strength of the material, and *f* the damage parameter relating to void fraction.

For the application of the GTN model embedded in ABAQUS, we need to specify the elastic and the hardening behavior of gradient grain size nano-grained copper, respectively. Here, we take $E=107\,GPa$ and $\nu =0.33$ as the elastic parameters and density of Cu is $\,\rho =8.9\,g/c{m}^{3}$. We specify the yield strength of individual grains in the gradient Cu samples by using the Hall-Petch relationship^25, 26^. Those parameters are abstracted from experimental results for gradient Cu^5, 6^, i.e.

2 $${\sigma }_{y}=63\,MPa+\frac{94.4\,MPa\sqrt{\mu m}}{\sqrt{d}\sqrt{\mu m}}MPa$$

where ${\sigma }_{y}$ and $d$ are the yielding strength and the grain size (in microns), respectively. We further assume that the ultimate strength ${\sigma }_{u}$ is also size-dependent, and we assign the ultimate strength of individual grains by the particular equation of

3 $${\sigma }_{u}=225\,MPa+\frac{70.04\,MPa\sqrt{\mu m}}{\sqrt{d}\sqrt{\mu m}}MPa$$

The ultimate strength occurs at a particular strain, and that strain has to be defined as well. Here we assume that the particular strain corresponding to the ultimate strength follows an inverse Hall-Petch relationship as

4 $${\varepsilon }_{u}=-0.124+0.848\sqrt{d}\sqrt{\mu m}$$

When a material point in a grain is strained to $\,{\varepsilon }_{u}$, strain softening occurs and the GTN model will capture the subsequent deformation. There are nine parameters to be specified in the GTN model which embedded in ABAQUS^24^. We further assume that the initial void fraction *f*_o_ in gradient Cu is inversely proportional to the square root of the grain size *d*. The following fitting formula is obtained:

5 $${f}_{0}=-5.474\times {10}^{-3}+\frac{3.237\times {10}^{-3}}{\sqrt{d}\sqrt{\mu m}}$$

It reflects the factor that the small grains were subjected to severe pre-plastic deformation, which could give rise to higher void fraction. Other material parameters of GTN model are tabulated in Table 1.

**Table 1:** Material parameters used in the Gurson-Tvergaard-Needleman model to capture the stress-strain curve of the gradient grain size structure.

*q*_1_	*q*_2_	*q*_3_	${\varepsilon }_{N}$	*S*_*N*_	*f*_*N*_	*f*_*C*_	*f*_*f*_
1.5	1	2.25	0.3	0.1	0.032	0.035	0.04

By using these parameters listed in Table 1, we are able to capture the engineering tensile stress-strain curves of the positive gradient (P-Gradient) structure and the negative gradient (N-Gradient) structure. The 3D geometrical model of the gradient sample has dimensions of $250\,nm(x)\times 250\,nm(y)\times 535\,nm(z)$ along x, y, and z direction, respectively. It consists of 221 grains. The grain size increases gradually from about $25\,nm$ at $z=535\,nm$ to about $250\,nm$ at $z=0\,nm$. The overall distribution of grain size shows good agreement with experimental results^27, 28^. The meshed model has 144697 linear tetrahedral elements, as seen in Fig. 1a. According to the assumption of a one-eighth model in ABAQUS, the numerical uniaxial tension tests were simulated by imposing Dirichlet boundary conditions on a vertex from the bottom of the model and three mutually perpendicular surfaces that are in contact with the vertex.

## Results and Discussion

What we show in Fig. 1a is the mesh of the one-eighth corner of a rectangular sample. By tuning the boundary condition of the structure in Fig. 1a, we can simulate two different types of gradient structure. The x-axis degree of freedom (DOF) of nodes in the $x=0\,nm$ plane and y-DOF of nodes in the $y=0\,nm$ planes are fixed. When the z-axis degree of freedom (DOF) of nodes in the $z=0\,nm$ plane is fixed, grains closer to the surface of the sample are finer, and we call the sample has positive gradient (P-Gradient). If the z-axis degree of freedom (DOF) of nodes in the $z=535\,nm$ plane is fixed, grains nearby the surface are larger than those in the core, and the structure is termed as negative gradient (N-Gradient). Figure 1b shows the simulated engineering stress-strain curve from the 3D model with a gradient structure under the uniaxial tension. The failure strain is also captured successfully. As a comparison, we also plot the engineering stress-strain curves from two homogenous materials of the free-standing surface-layer with grain size 25 *nm* and the free-standing core-layer with grain size 250 *nm* by using the same 3D gradient model (P-Gradient). The simulated results of gradient hierarchical structure show a yielding strength of about $370.3\,MPa$ and a uniform elongation of about 35%. The yield strength of the gradient hierarchical structure is consistent with the mixing law:

6 $${\bar{\sigma }}_{y}=\sum _{i=1}^{n}{f}_{i}{\sigma }_{yi}$$

where, *i* refers to grains in the i-th layer, and $n$ is the total number of layers, *f*_i_ and ${\sigma }_{yi}$ are the volume fraction and the yield strength of a layer, respectively. The yield strength is about 50% percentage higher than that of the core material with an average grain size of $250\,nm$; there is no reduction in ductility. Such an extraordinary mechanical performance of gradient grain size structure is analogous to that reported in experimental observation^5^. These results reveal an effective way which results in a suppression of the strain localization to evade the strength-ductility trade-off by using a gradient structure.

**Figure 2:**
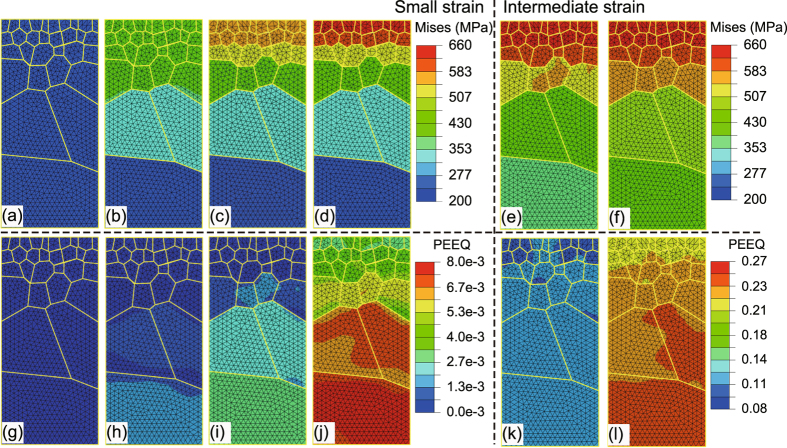
Contours of von Mises stress and equivalent plastic strain of the P-Gradient structure at strains keyed in Fig. 1b. (**a**) to (**f**) Von Mises stress contours of the P-Gradient structure at (I) to (VI), in turn. (**g**) to (**l**) The corresponding equivalent plastic strain contours of the P-Gradient structure.

In Fig. 1b, we keyed six representative status at various applied true strains, with a blow-out of the portion near the yielding point. To explain why a gradient grain size structure is so beneficial for strength and tensile ductility, we show the contours of gradient von Mises stress of the six representative points at different applied strains from the cross section perpendicular to y-axis. Figure 2a–f shows the evolution progress of the spatial distributions of Mises stress. From the beginning of the deformation progress to the strain of 0.25% (Fig. 2a), at this stage, the deformation progress is still in linear elastic, and the spatial distribution of the Mises stress is totally uniform. As the strain increases to 0.98% (as shown in Fig. 2b–d), the difference in Mises stress between the surface-layer and the core-layer increases dramatically because the plastic deformation initiates from the core-layer and propagates to the surface region. When the applied strain changes from 0.98% to 24.31%, as shown in Fig. 2d–f, the progressive softening starts from the surface-layer to the core-layer; the difference of stress between the core-layer and the surface-layer decreases gradually.

**Figure 3:**
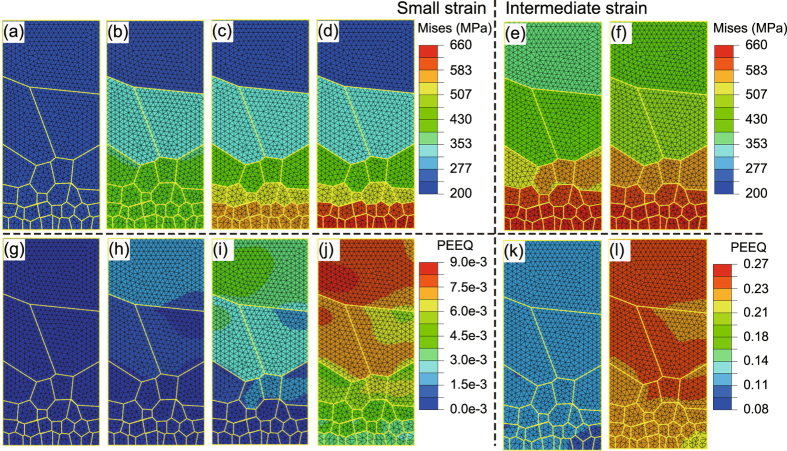
Contours of von Mises stress and equivalent plastic strain of the N-Gradient structure at strains keyed in Fig. 1b. (**a**) to (**f**) Von Mises stress contours of the N-Gradient structure at (I) to (VI), in turn. (**g**) to (**l**) The corresponding equivalent plastic strain contours of the N-Gradient structure.

We further plot the contours of equivalent plastic strain at these representative points. The spatial distribution of equivalent plastic strain also shows a gradient: As the applied strain increase, the value of equivalent plastic strain is smaller in the surface region than that in the core, as shown in Fig. 2g–j. When the applied strain changes from 0.98% to 24.31%, the gradient of spatial distribution of equivalent plastic strain trend to be uniform as seen in Fig. 2j–l. We show in Fig. 3 both the contours of stress and equivalent plastic strain of the N-Gradient structure. The evolution process is similar to the P-Gradient structure; the stress and strain distributions in this case are opposite to those of the P-Gradient distribution, as previously described.

**Figure 4:**
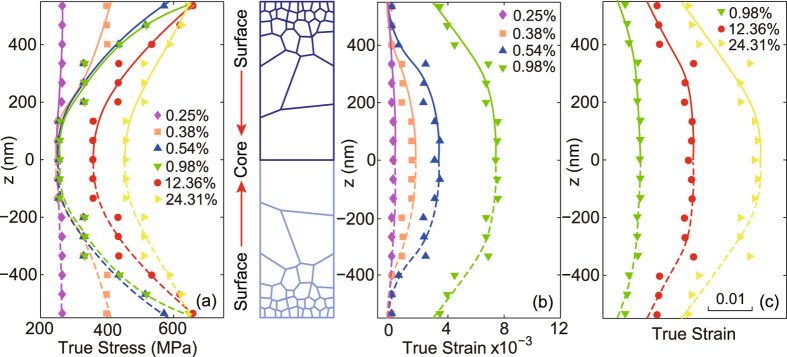
Distributions of von Mises stress and equivalent plastic strain in the cross section of P-Gradient structure at strains keyed in Fig. 1b. (**a**) Von Mises stress distribution of the P-Gradient structure. (**b**) and (**c**) Equivalent plastic strain distribution of the P-Gradient structure.

We now examine closely the partition of stress and strain along the gradient direction (the z-axis) at different applied true strain. We divide the sample along the z-axis by 9 equal-width slices and calculate the volumetric average of the stress and strain within each region. The variation in Mises stress along the gradient direction for P-Gradient sample is shown in Fig. 4a. For better view, we plot the stress in the whole sample by taking its symmetry into consideration. At small strain, the distribution of Mises stress is nearly uniform due to initial elastic deformation of all these grains. With the increase of strain, large grains yield first, and the plastic front propagates from the soft core to the hard surface in P-Gradient sample, as evidently seen in the large equivalent plastic strain in the core, as demonstrated in Fig. 4b. With the gradual increasing from small applied strain (e.g., 0.98% of the green curve) to larger applied strain (e.g., 12.36% of the red curve), the surface region nearly reaches the ultimate strength, resulting in a slow increase of the Mises stress (see Fig. 4a), In contrast, the core region undergo a hardening progress which lead to the dramatic increase of Mises stress. When the applied strain reaches about 24.3% (as shown with the yellow curve), due to the appearance of a softening progress, the Mises stress of the surface-layer begin to decrease slightly. Meanwhile, the stress-strain response in the core region is still in the hardening stage which leads to the steady increase of the Mises stress. According to the strain distribution in Fig. 4c, we note that the core region with big grain size exhibits the maximum plastic strain level.

**Figure 5:**
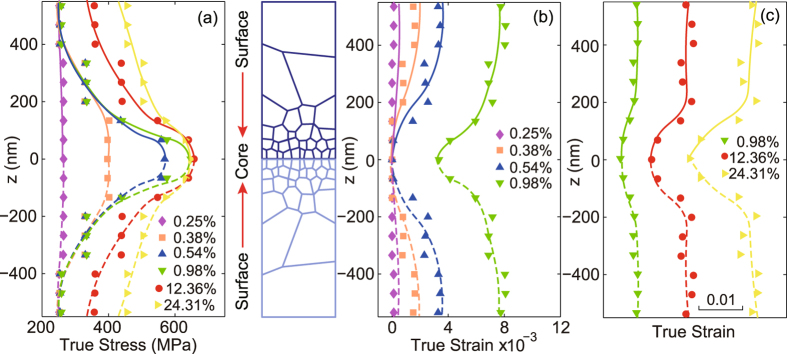
Distributions of von Mises stress and equivalent plastic strain in the cross section of N-Gradient structure at strains keyed in Fig. 1b. (**a**) Von Mises stress distribution of the N-Gradient structure. (**b**) and (**c**) Equivalent plastic strain distribution of the N-Gradient structure.

The stress and equivalent plastic strain distribution in the N-Gradient sample with hard core and soft surface are also explored, as shown in Fig. 5. In contrast to the P-Gradient sample, the distribution of stress and equivalent plastic strain are reversed the spatial coordinate. The fact that soft region yields first and carries more plastic deformation at the late stage remains the same as seen in Fig. 4 for the P-Gradient sample. It is worth noting that, with the increase of the applied strain, the difference of equivalent plastic strain between the soft region and the hard region in both types of samples increases gradually, but is within 1–2% to its maximum level, and the exact value is primarily determined by the difference of the flow stress in those regions.

In both types of three-dimensional samples, we demonstrate an effective deformation mechanism – hard shell (fine-grained layer) carry more stress and soft core (coarse-grained layer) share more plastic deformation which breaks the limitations of the ductility of the homogeneous constitutive model. These simulated results show a unique feature of the gradient structure under the uniaxial tension test, compared with those non-uniform deformation experimental test e.g., torsion, bending and indentation from previous research^29–32^.

**Figure 6:**
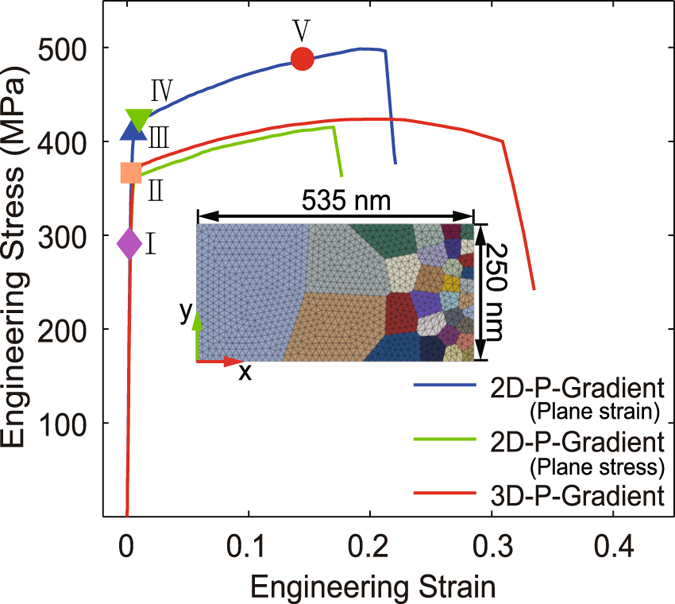
Quasi-static axial tensile engineering stress-strain curves for the two-dimensional and three-dimensional FEM model with positive grain size gradient (P-Gradient). The inset is for the dimensions of two-dimensional model used here. Five representative applied strains are marked in this figure, point I to V are the applied true strain 0.25%, 0.38%, 0.54%, 0.98% and 12.36%, respectively.

**Figure 7:**
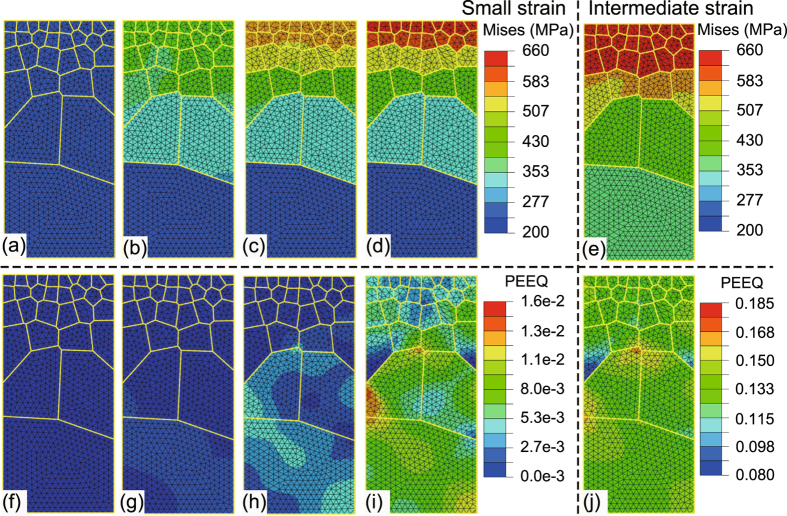
Contours of von Mises stress and equivalent plastic strain from the two-dimensional plane strain sample. (**a**) to (**e**) Von Mises stress contours of the P-Gradient structure at points (I) to (V) keyed in Fig. 6, in turn. (**f**) to (**j**) The corresponding equivalent plastic strain contours.

**Figure 8:**
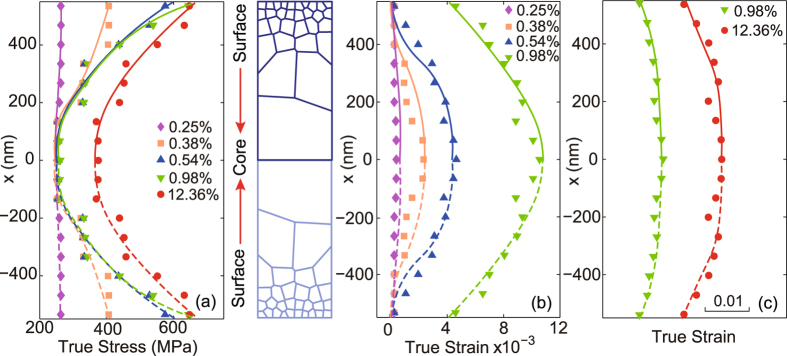
Distributions of von Mises stress and equivalent plastic strain in the two-dimensional plane strain sample with P-Gradient. (**a**) Von Mises stress distribution at different strains. (**b**) and (**c**) Equivalent plastic strain distribution at corresponding strains.

In addition, comparative analysis is made between 3D model and 2D model (both plane stress and plane strain condition) with the same constitutive model, mesh density, boundary condition and positive gradient structure, as shown in Figs 6 to 8. Both 2D and 3D gradient structures capture the stress gradient and strain gradient due to the presence of structure gradient (Figs 7 and 8). In terms of ductility, the influence of dimension is significant, as evidently seen in Fig. 6. It is due to the factor that 2D systems are more sensitive to local heterogeneity and lead to premature localization (Fig. 7). Due to greater deformability, 3D systems exhibit larger strain gradient (Fig. 4) at the late stage of deformation in contrast to that of 2D samples (Fig. 8).

## Summary and conclusions

In conclusion, we explored the deformation mechanism of the gradient structure material by using a 3D model that considers the grain size-dependence effect. By applying the uniaxial tension on the 3D gradient crystalline model, we showed the contours and distribution curves of von Mises stress and equivalent plastic strain. The results revealed that when the applied strain is at a low level, these spatial gradient contours arise due to the progressive yield progress of different grain size, and coarse grains in the core region have lower Mises stress and higher equivalent plastic strain, compared to fine grains in the surface layer i.e. the fine-grained layer carry more stress and the coarse-grained layer share more plastic deformation. As the applied strain gradually increases to higher level, the gradient of Mises stress and equivalent plastic strain are trend to be uniform, but there is still a difference between the distribution of surface and the one of core. Our simulations convincingly demonstrated the yielding strength of the hierarchical structure can be significantly enhanced without the obvious ductility loss for a gradient structure. The introduced deformation mechanism of gradient structure could be broadly employed to enhance the strength and retain the tensile ductility of metallic materials. In addition, the compared result reveals that 3D model may better reflect plastic deformation gradient and can be used to deepen our understanding of deformation behavior in gradient structures.

### Data Availability

All data generated or analysed during this study are included in this published article.

## References

[1] Ray AK, Mondal S (2005). Bamboo—A functionally graded composite-correlation between microstructure and mechanical strength. J. Mater. Sci..

[2] Gao H, Ji B (2003). Materials become insensitive to flaws at nanoscale: Lessons from nature. Proc. Natl. Acad. Sci..

[3] Bruet BJ, Song J (2008). Materials design principles of ancient fish armour. Nat. Mater..

[4] Lu KN (2014). Making strong nanomaterials ductile with gradients. Science.

[5] Fang TH, Li WL (2011). Revealing extraordinary intrinsic tensile plasticity in gradient nano-grained copper. Science.

[6] Fang TH, Tao NR, Lu K (2014). Tension-induced softening and hardening in gradient nano-grained surface layer in copper. Scripta Mater..

[7] Chen W, You ZS (2017). Mechanically-induced grain coarsening in gradient nano-grained copper. Acta Mater..

[8] Wu X, Jiang P (2014). Extraordinary strain hardening by gradient structure. Proc. Natl. Acad. Sci..

[9] Wei Y, Li Y (2014). Evading the strength–ductility trade-off dilemma in steel through gradient hierarchical nanotwins. Nat. Commun..

[10] Ma Z, Liu J (2016). Strength gradient enhances fatigue resistance of steels. Sci. Rep-UK..

[11] Liu Y, Wei Y (2016). Gradient driven anomalous reversible plasticity in conventional magnesium alloys. Extreme Mech. Lett..

[12] Lee JK, Ehrlich FR (1988). An analysis for the effect of a grain size gradient on torsional and tensile properties. Metall. Mater. Trans. A.

[13] Li J, Soh AK (2012). Modeling of the plastic deformation of nanostructured materials with grain size gradient. Int. J. Plast..

[14] Li J, Soh AK (2012). Enhanced ductility of surface nano-crystallized materials by modulating grain size gradient. Model. Simul. Mater. Sc..

[15] Zeng Z, Li X (2016). Gradient plasticity in gradient nano-grained metals. Extreme Mech. Lett..

[16] Gurson AL (1977). Continuum theory of ductile rupture by void nucleation and growth: path i-yield function and flow rules for porous ductile media. J. Eng. Mater.-T. ASME.

[17] Tvergaard V (1981). Influence of voids on shear band instabilities under plane strain conditions. Int. J. Fract..

[18] Tvergaard V (1982). On localization in ductile materials containing spherical voids. Int. J. Fract..

[19] Tvergaard V, Needleman A (1984). Analysis of the cup-cone fracture in a round tensile bar. Acta Metall..

[20] Chu CC, Needleman A (1980). Void nucleation effects in biaxially stretched sheets. J. Eng. Mater. Technol..

[21] Li H, Fu MW (2011). Ductile fracture: Experiments and computations. Int. J. Plast..

[22] Nahshon K, Xue Z (2009). A modified Gurson model and its application to punch-out experiments. Eng. Fract. Mech..

[23] Celentano DJ, Chaboche JL (2007). Experimental and numerical characterization of damage evolution in steels. Int. J. Plast..

[24] Abaqus FEA, D.S.S.D. Systèmes, Editor 2007.

[25] Hall EO (1951). The Deformation and Ageing of Mild Steel: III Discussion of Results. Proc. Phys. Soc. Lond. B.

[26] Petch NJ (1953). The Cleavage strength of polycrystals. J Iron Steel Inst.

[27] Zhu T, Li J (2009). Mechanics of ultra-strength materials. MRS Bull..

[28] Yip S (1998). The strongest size. Nature.

[29] Rice JR (1976). In *Proc. of the 14th Int. Congress on ‘Theoretical and Applied Mechanics’. Delft.*. North-Holland Publishing Co..

[30] Fleck NA, Muller GM (1994). Strain gradient plasticity: Theory and experiment. Acta Metall. Et Mater..

[31] Evans AG, Hutchinson JW (2009). A critical assessment of theories of strain gradient plasticity. Acta Mater.

[32] Nix, W. D. & Gao, H. Indentation size effects in crystalline materials: A law for strain gradient plasticity. *J Mech. Phys. Solids* **46**(3), 411–425 (1998).

